# Rapid and robust spatiotemporal dynamics of the first-order phase transition in crystals of the organic-inorganic perovskite (C_12_H_25_NH_3_)_2_PbI_4_

**DOI:** 10.1038/srep16634

**Published:** 2015-11-16

**Authors:** Aymen Yangui, Mouhamadou Sy, Liang Li, Younes Abid, Panče Naumov, Kamel Boukheddaden

**Affiliations:** 1 Groupe d’Etudes de la Matière Condensée, Université de Versailles, CNRS UMR 8635, 45 Avenue des Etats Unis, 78035 Versailles, France; 2 Laboratoire de Physique Appliquée, Faculté des Sciences de Sfax, Route de Soukra km 3.5 3000 Sfax, Tunisia; 3 New York University Abu Dhabi, P.O. Box 129188, Abu Dhabi, United Arab Emirates

## Abstract

The dynamics of the thermally induced first-order structural phase transition in a high-quality single crystal of the organic-inorganic perovskite (C_12_H_25_NH_3_)_2_PbI_4_ was investigated by optical microscopy. The propagation of the straight phase front (habit plane) during the phase transition along the cooling and heating pathways of the thermal hysteresis was observed. The thermochromic character of the transition allowed monitoring of the thermal dependence of average optical density and aided the visualization of the interface propagation. The thermal hysteresis loop is 10 K wide, and the interface velocity is constant at *V* ≈ 1.6 mm s^–1^. The transition is accompanied with sizeable change in crystal size, with elongation of ~6% along the *b* axis and compression of ~ –2% along the *a* axis, in excellent agreement with previously reported X-ray diffraction data. The progression of the habit plane is at least 160 times faster than in spin-crossover materials, and opens new prospects for organic-inorganic perovskites as solid switching materials. Moreover, the crystals of (C_12_H_25_NH_3_)_2_PbI_4_ are unusually mechanically robust and present excellent resilience to thermal cycling. These hitherto unrecognized properties turn this and possibly similar hybrid perovskites into perspective candidates as active medium for microscopic actuation.

Organic-inorganic perovskite materials based on metal halides have attracted attention recently due to their potentials in applications as light-emitting materials in electroluminescent devices^1^,^2^. The applications of such materials include the development of electronic^3^ and optoelectronic devices^4^, and they are also being considered as light-harvesting media in solar cells^5^. Since 2013 the hybrid perovskites have been studied extensively for their high conversion efficiencies within the quest for next-generation photovoltaic devices^6^. Particularly, two- and three-dimensional organic-inorganic perovskites are prospective materials because they exhibit multiple phase transitions^7^,^8^,^9^ that could considerably affect their photoluminescence properties^10^ and the performance in solar cells^11^,^12^,^13^. Most of these thermal transformations are first order phase transitions between low-temperature (LT) and high-temperature (HT) phases with well-characterized physical properties. The switching between phases can be monitored by a variety of analytical techniques because of the large changes in physical parameters (structure, volume, color, specific heat, etc.). The occurrence of first order transition with hysteresis loop in these materials is normally attributed to thermally-induced structural reorganization of the organic chains located between the inorganic layers: the organic chains are ordered bellow the transition temperature and as they become disordered in the HT phase they drive change in crystal symmetry. As the rotational disorder increases with increasing temperature, the interlayer spacing decreases, resulting in local collapse of the structure in the direction perpendicular to the inorganic sheets^14^,^15^. However, the dynamics of these phase transitions is yet to be clarified. The spatiotemporal characteristics of the phase transitions are essential to understand the ensuing effects on their performance as they are directly related to the mechanistic details of the switching of their electronic properties.

We report here the first visualization and quantitative analysis of the spatiotemporal behavior of the first order phase transition in the organic-inorganic perovskite material (C_12_H_25_NH_3_)_2_PbI_4_ (hereafter abbreviated as C_12_PbI_4_)^16^,^17^,^18^ that was accomplished by using optical microscopy. The crystal structure of this material has been described as a two-dimensional (2D) perovskite structure that comprises inorganic sheets of PbI_4_ units that alternate with double layers of the organic cation, C_12_H_25_NH_3_^+^ (Fig. S1 in the Supporting Information, SI). The organic and inorganic moieties are connected by hydrogen bonds. Upon heating, the symmetry of C_12_PbI_4_ changes from orthorhombic (*Pbca*) to monoclinic (*P*2_1_/*a*). This transformation is accompanied by color change at 317.5 K from yellow to orange. On cooling, the LT phase is recovered below 307.5 K. Thus, this change is related to a first-order phase transition accompanied with thermal hysteresis^9^. Here, we focus on the spatiotemporal features of the first order transition in C_12_PbI_4_ in a study that aims to clarify its macroscopic mechanism, particularly in respect to a single- *vs* multi-droplet domain nucleation mechanism, as well as on its progression at a microscopic scale.

Thin, elongated microcrystal of C_12_PbI_4_ was investigated by optical microscopy. At room temperature, where the crystal existed in the LT phase, it measured 20 μm (*L*_a_) × 186 μm (*L*_b_) × 5 μm (*L*_c_). A set of typical images recorded during the transformation is shown in Fig. 1 for one crystal orientation where the crystallographic axis *c* is parallel to the microscope axis. The heating and cooling rates were fixed at 0.2 K min^–1^. During the transition from the LT phase to the HT phase, a formation of a well-defined straight front (interface) between the two phases of different color was observed that propagated through the entire crystal at a constant velocity of about 1.6 mm s^–1^ (see below). The transformation was completed within ~120 ms. According to the value of the heating rate, we estimated that the crystal temperature had increased by about 0.37 K during the transformation (the real value is probably lower due to the latent heat of the reaction). Therefore, we can safely consider that the snapshots in Fig. 1 describe an isothermal process.

When subject to heating-cooling cycles, the C_12_PbI_4_ microcrystal displays color change from yellow to orange on heating, followed by change from orange to the initial yellow color on cooling (Fig. 1a). Movies S1–S3 in the Supporting Information show this spatiotemporal transformation on two different single crystals. The observed color change is consistent with the first order phase transition reported for C_12_PbI_4_^9^,^19^. The interface is almost parallel to the *a* axis and propagates along the *b* axis of the unit cell. The snapshots of the crystal at different temperatures in Fig. 1a show that the transformation is accompanied by significant change in crystal size. Upon heating to around 317.5 K, the crystal shows sizable length expansion of ~6% along the *b* axis, from 186 μm to 197 μm. Concomitantly, the crystal width (along the *a* axis) contracts by 2%, from 20 μm to 19.6 μm. On cooling, this transformation is reversible and the crystal reverts to its initial size and shape around 307.5 K. We recorded the thermal variation of the length (*L*_b_) and width (*L*_a_) of the crystal within the thermal hysteresis. The results are summarized in Fig. 2, where the temperature (*T*) variation of the relative length and width is shown as ∆*L*_b_/*L*_b_ = [*L*_b_(*T*)-*L*_b_(LT)]/*L*_b_(LT) and ∆*L*_a_/*L*_a_ = [*L*_a_(*T*)–*L*_a_(LT)]/*L*_a_(LT) (note that, regretably, two-dimensional optical microscopy does not give an access to the change in the thickness (∆*L*_c_/*L*_c_) of the material). A thermal hysteresis loop, centered at *T*_0_ = 312.5 K with width Δ*T* = 10 K was observed in both cases, in line with the first-order character of the transition.

It is important to note that the width of the thermal hysteresis can depend significantly on the temperature sweep rate; higher scan rates increase the width of the thermal hysteresis and cause its distortion. The high- and low-temperature branches respond to this change in conditions at different timescales. If the middle of the hysteresis (*T*_0_ = 312.5 K) is considered as the Maxwell point of the transition, then starting from the LT or HT phase and changing the temperature away from *T*_0_ by heating or cooling, respectively, will cause switching of the system to a metastable state. The lifetimes *τ*(*T*) of these metastable states, which are infinite at *T*_0_, become finite and decrease significantly following a power law of |*T* – *T*_0_| due to decrease of energy barriers of nucleation (for details, see ref. 20). Once the nucleation of the stable phase has started and the phase has grown over a critical size, the system becomes unstable and the transformation process switches from stochastic to cooperatively deterministic. In the current study, these phenomena take place at the transitions temperatures *T*^+^ = 317.5 K and *T*^−^ = 307.5 K. For fast heating or cooling sweep rates, respective superheated or supercooled states are reached, which shifts the limiting temperatures *T*^±^ (=*T*_tr_) and increases the width of the hysteresis.

Table 1 summarizes the crystallographic details^9^ of the HT (*T* = 319 K) and LT (*T* = 293 K) phases for a single crystal of the title material. On heating, the *b* axis increases from 8.5149(6) Å to 9.0031(2) Å and the *a* axis shrinks from 8.8645(1) Å to 8.6882(6) Å, giving relative changes ∆*b*/*b* = 5.7% and ∆*a*/*a* = –2.0%. These results are consistent with the optical microscopy results. According to the crystallographic data in Table 1, during the transition the crystal undergoes strong change in thickness. This is paralleled by strong change in the density of the material. The transformation is also accompanied by a significant change in optical absorption (Fig. 1b) that is visually readily observed as photochromism and facilitates the observation of the phase progression by optical microscopy.

The presence of intense excitonic absorption band around 489 nm (2.55 eV) and the notable shift in the optical absorption maxima during the transition (Fig. 1b) were used together with the colorimetric method introduced in ref. 21 to monitor the structural transformation by the “green pixels” of the optical density (OD). Figure 3a depicts the thermal dependence of the spatially averaged OD of the crystal during heating and cooling. The hysteresis loop of 10 K is in accordance with the value determined from the change in crystal size (Fig. 2).

During the transition (induced either by heating or cooling) the transition onset was observed as a single macroscopic domain that grows and propagates throughout the whole crystal (Fig. 1 and SI Movies S1–S3). Upon heating, the nucleation starts at the tip of the crystal (shown as the left terminus of the crystal shown in Fig. 1a). Figure 3b shows the time dependence of the interface position. The linearity of the plot indicates that the front propagates at constant velocity, determined as *V* ≈ 1.6 mm s^–1^. The front orientation appears almost perpendicular to the *b* axis of the crystal, however detailed inspection (on several single crystals) revealed a tilt angle of ~3° with respect to the direction normal to the *b* axis. On cooling, the front evolves at the opposite end (relative to the start), as shown on the right terminus of the crystal in Fig. 1a, and propagates with approximately identical speed as the forward transition.

Phenomenologically, there are two factors that affect the velocity (*V*) of front transformation, the driving force and the absolute temperature. The former is determined by the difference between the free energies of the phases; *V* is zero at *T*_0_ ~312.5 K and increases as *T*_tr_ shifts away from *T*_0_ in either direction. The absolute temperature factor, on the other hand, affects *V* monotonously; higher *T*_tr_ is related to higher *V*. However, since *T*_tr_ increases with the temperature scan rate (see SI), the interface velocity is expected to be an increasing function of the temperature sweep rate. However, the response of the system to the fast thermal rate is not only limited by the experimental setup for temperature control (electronics of the temperature controller, heater, cold finger and exchange gas) but also by the heat diffusion in the crystal, which is intrinsic property of the material. In a material of length *L* with a heat diffusion constant *D* the velocity of heat diffusion is *V*_*th*_ ~ *D*/*L^2^*. If we consider that the hybrid perovskites have thermal diffusion constants similar to those of polymers, which are normally in the range 200-300 μm^2^ s^-1^, *V*_th_ is estimated to ~1.5 mm s^-1^, which is surprisingly close to the measured experimental velocities of front propagation. However, it should be noted that although driven by temperature, the interface propagation also has an intrinsic rate that is governed by specific changes of the elastic medium (anisotropy, relative volume changes, etc.). These observations are reminiscent of the front propagation dynamics in spin-crossover (SC) single crystals^21^,^22^,^23^,^24^ where the local volume changes are delocalized over several cell parameters, thereby generating the long-range character of the interaction. In SC materials, which have similar values of thermal diffusions, similar sweep rates lead to interfaces propagating at velocities < 10 μm s^-1^. However, two fundamental differences between the behavior of the two types of molecular solids should be noted. First, the typical velocities of interface progression in SC solids are almost hundred/thousand times smaller (typically on the order of μm s^–1^) relative to those recorded here. Second, the interface velocities during cooling and heating in SC solids are significantly different (two to three times faster on heating), while they are identical in the material studied here. This difference is attributed to the low Debye temperature (*θ*_D_ ≈ 140 K) of the perovskite materials^25^ which have ductile abilities for extensive deformation in both states, compared to SC solids (*θ*_D_ ≈ 220 K) whose rigidity is strongly affected by the phase transition^26^.

The spatial profile of the interface could provide additional information on the origin of this fast transition. The profile of the interface was analyzed at different time points by plotting the optical density along a straight line parallel to the *b* axis (the propagation direction) and across the interface between the two phases. The profiles were sampled at 0.01 s-intervals. The plot of successive profiles in Fig. 4a shows that the shape of the interface is invariant and is ~2 μm wide. An interesting feature are the small variations in optical density of comparable amplitude at either side of the LT/HT interface (Fig. 4a). This effect can be attributed to variation in the refractive index of the crystal in the vicinity of the progressing front and are indicative of mechanical stresses that cause light scattering. Figure 4b shows the average OD profile averaged after all curves were offset to overlap so that their center is fixed at *x* = 110 μm. This average curve was analyzed using the propagating solutions of the Kolmogorov’s equation^27^, given as


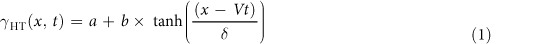


where *γ*_HT_(*x*, *t*) = [OD(*x*, *t*) – OD(LT)]/[OD(HT) – OD(LT)] is the fraction of the HT phase at position *x* and time *t*, *V* is the interface propagation speed, *δ* is the interface width, and *a* and *b* are constants. When fitted by the model, the experimental curve yielded the following parameters: *V* = 1.60(1) mm s^–1^, *δ* = 2.05(2) μm, *a* = 0.28(1) and *b* = –0.16(2).

To address the problem of local kinetics of the transformation process, the change of OD along the *a* axis with time was extracted during the front propagation, that is, perpendicular to the front propagation direction. The plot of the OD as a function of time provided a set of kinetic curves whose average (Fig. 5) can be described in terms of a nucleation-and-growth process, following the well-known KJMA-type law^28^:


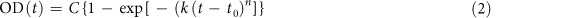


The best fit of the experimental data in Fig. 5 afforded the following parameters: *t*_0_ = 5.981(18) s, *C* = 0.994(16), *k* = 2.20(21) s^–1^, and *n* = 1.974(29). These results provide the local time scale of the nucleation process that precedes the front, which was estimated to 1/*k* ~ 0.5 s. The value of the Avrami exponent (*n*) describes the dimensionality of the nucleation process. Its value is close to 2, suggesting a lamellar process, which is consistent with the 2D structure of the material.

The accurate determination of the progression of the habit plane in C_12_PbI_4_ allows direct comparison with other phase transitions. The SC transitions are a convenient benchmark to that end since accurate data on these systems are available in the literature from measurements performed by optical microscopy and within the prospects for their utility as switching and memory-storing materials. Comparison of the progression rate of the habit plane with the SC materials (Table 2) showed that the phase change in the hybrid perovskite studied here is at least 160 times faster than SC transitions! Yet, the crystals of C_12_PbI_4_ display another trait that is advantageous to the SC systems. The small (normalized) volume change of this perovskite whose unit cell is doubled at the transition temperature on cooling (*Z* = 2 in the HT phase, *Z* = 4 in the LT phase) of ~0.9% turns the structure mechanically very robust; the crystals can be switched back and forth between the two phases without any apparent deterioration. On the contrary, the SC transitions are accompanied by a relative unit cell change of ~3–4%. An important characteristic that reinforces this resilience is the anisotropy of the structural deformation. The changes of the unit cell along the crystallographic axes in C_12_PbI_4_ during the phase transition on heating are anisotropic, with ∆*a*/*a* ~ −2%, ∆*b*/*b* ~ 6%, and ∆*c*/*c* ~ −3% (note that *Z* = 4 in the LT phase). The 2D structure of this material, the mechanical flexibility of the organic chains and the shrinking of the unit cell along *a* and *c* axes on heating, channel a part of the transformational stress which could have caused dislocations or fractures in the material if the expansions along the three axes had been of same sign. An isotropic local deformation as in the case of most SC systems, would have been detrimental. In most SC crystals, this results in dislocations that release the long-range elastic strain (a mismatch-free interface was possible in only one case of a SC single crystal that shows anisotropic deformation of the unit cell)^24^. On the other hand, the spin-crossover solids appear to be much more ductile. As a consequence, most of the crystals of SC materials are brittle^29^,^30^ and their integrity is affected by the large isotropic change in unit cell volume. Indeed, with exception of two cases (a two-step incomplete transition^31^ and anisotropic deformation of the unit cell^23^), this setback of the SC solids has been the main obstacle to direct observation of the phase transition in their single crystals.

The origin of the different rates of phase transition between C_12_PbI_4_ and the SC solids can be traced back to the interplay between three factors: (a) temperature, (b) driving force arising from the difference in the free energies of the two phases and (c) the rate of local nucleation process (*k*) that appears in Eq. 2. The interface velocity is zero at the equilibrium transition temperature, *T*_0_ (the Maxwell point corresponding to equal free energies of the two phases)^23^ and increases as *T* moves away from *T*_0_ in either direction. Maximum velocity is observed at the limiting (upper and lower) temperatures of the hysteresis loop^23^. Figure S2 (SI) shows the variation of the free energy with the order parameter inside the thermal hysteresis loop. The height of the dynamic barrier is directly derived from the double-well shaped curves, from where the stable, metastable and unstable states are easily identified at each temperature. The heights of the (macroscopic) barriers, ∆*E*_HL_ and ∆*E*_LH_, govern the lifetimes, *τ*_HL_ and *τ*_LH_, of the associated metastable states that are proportional to exp(*β*∆*E*_HL_) and exp(*β*∆*E*_LH_), respectively.

The front velocity *V* is the net result of two opposite processes that are proportional to the inverse lifetimes of the HL and LT states and to the inverse local time scale of the nucleation process (1/*k*):


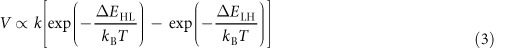


It should be noted that the values of all factors in Eq. 3 are higher for the perovskite materials. Indeed, their enthalpy change at the transition (∆*H* ≈ 0.5 kJ mol^–1^) is smaller than those of the SC materials (∆*H* ≈ 7 kJ mol^–1^). Moreover, their transition temperatures are also higher. The local nucleation rate in C_12_PbI_4_, *k* ≈ 2 s^–1^ is about 75 times higher than that of [Fe(btr)_2_(NCS)_2_]·H_2_O (‘btr’ stands for bistriazole) (Table 2), *k* = 0.029 s^–1^ (0.0069 s^–1^ from X-ray diffraction^32^).

The KJMA equation (Eq. 2), from which *k* was derived sheds light on this difference. The equation describes formation of small “droplets” of the equilibrium phase in the (uniform) matrix of the metastable phase with a different unit cell volume. In case of isotropic and homogeneous medium, the elastic energy required to expand or shrink an element of volume 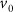 to volume 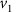 is given by the Eshelby relation:


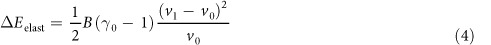


where *B* is the bulk modulus, which is about 15 GPa^25^ for hybrid perovskites and 25 GPa for SC^33^, *γ*_0_ (~3) is the Eshelby constant (the surface energy was omitted from Eq. 4). The volume change at the transition induces differences in the bulk moduli of the LT and HT phases. When analyzed within the frame of the Grüneisen approximation, considering the quadratic dependence of the bulk modulus *B* on the Debye frequency (or temperature), it leads to the following relation^34^:


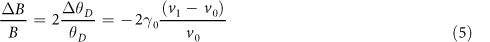


which shows that larger the relative volume change the stronger the relative bulk modulus change. As a result of the difference in the relative volume misfit, 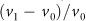, the elastic energy in Eq. 4, that corresponds to the barrier that a nucleus has to overcome, is lower for C_12_PbI_4_ relative to SC solids and drives faster nucleation. However, these considerations apply to isotropic deformations, and their extension to anisotropic cases is not straightfoward. The anisotropic change of unit cell parameters across the transition could effectively absorb the excess elastic strain generated by the lattice parameter misfit. Additionally, the deformation of the current system occurs with different signs along the unit cell axes (expansion along the *b* axis, shrinking along the *a* and *c* axes), which then reduces the strain along the brittle axis as well as the total volume change. The collective contribution of the above factors is central to the high speed and the mechanical robustness of this material. The reversibility of the phase transition in these materials paves the way not only to new applications as actuators, but also to tunable band-gap materials.

In summary, the real-time spatiotemporal dynamics of the first-order thermal transition in a two-dimensional organic-inorganic perovskite single crystal, C_12_PbI_4_, was visualized by optical microscopy. By using high-quality crystals we succeeded in capturing the transformation and the front dynamics during the phase transition by both heating and cooling. It was found that the first-order transformation proceeds with a straight interface that has stable orientation and shape, and propagates at a constant velocity of 1.6 mm s^–1^. This transition is two orders of magnitude faster than those reported for spin crossover single crystals^21^,^22^,^23^,^24^, although it is much slower compared to the transitions recorded in some organic thermosalient and photosalient solids (“jumping crystals”)^35^,^36^,^37^,^38^. During the phase transition on heating the crystal is elongated by ~6% but its width decreases by ~ –2%. This change was accompanied by color change from yellow to orange. The transformation is reversible. Analysis of the temporal dependence of the optical densities on heating and cooling confirmed the occurrence of a first order transition with a hysteresis loop of 10 K, which was also observed with the thermal behavior of the crystal size. Similar behavior has been reported with spin-crossover crystals [{Fe(NCSe)(py)_2_}_2_(μ-bpypz)]^39^ (py = pyridine and *m*-bpypz = 3,5-bis(2-pyridyl)-pyrazolate)^40^, and [Fe(bbtr)_3_](ClO_4_)_2_ (bbtr = 1,4-di(1,2,3-triazol-1-yl)butane)^41^, where the crystals stretch along one direction and shrink along the other during a high-spin-to-low-spin phase transition^24^. Interestingly, and contrary to various molecular single crystals that undergo first order transitions accompanied by volume change, the crystals of the material studied here are very robust and endowed by excellent mechanical resilience. Indeed, the crystals maintain their integrity even after several thermal cycles. The most probable reason for this appears to be the existence of a free strain interface. This interface emerges during the phase transition and effectively reduces the total elastic energy due to lattice mismatch between the LT and HT phases. Numerical simulations using adequate elastic models based on X-ray diffraction data of the LT and the HT phases are now in progress in our laboratories to gain deeper understanding on the various facets of this complex issue, and in particular on the physical mechanisms that govern the orientation of the interface.

## Additional Information

**How to cite this article**: Yangui, A. *et al.* Rapid and robust spatiotemporal dynamics of the first-order phase transition in crystals of the organic-inorganic perovskite (C_12_H_25_NH_3_)_2_PbI_4_. *Sci. Rep.* **5**, 16634; doi: 10.1038/srep16634 (2015).

## Supplementary Material

Supplementary Information

Supplementary Information

Supplementary Information

## Figures and Tables

**Figure 1:**
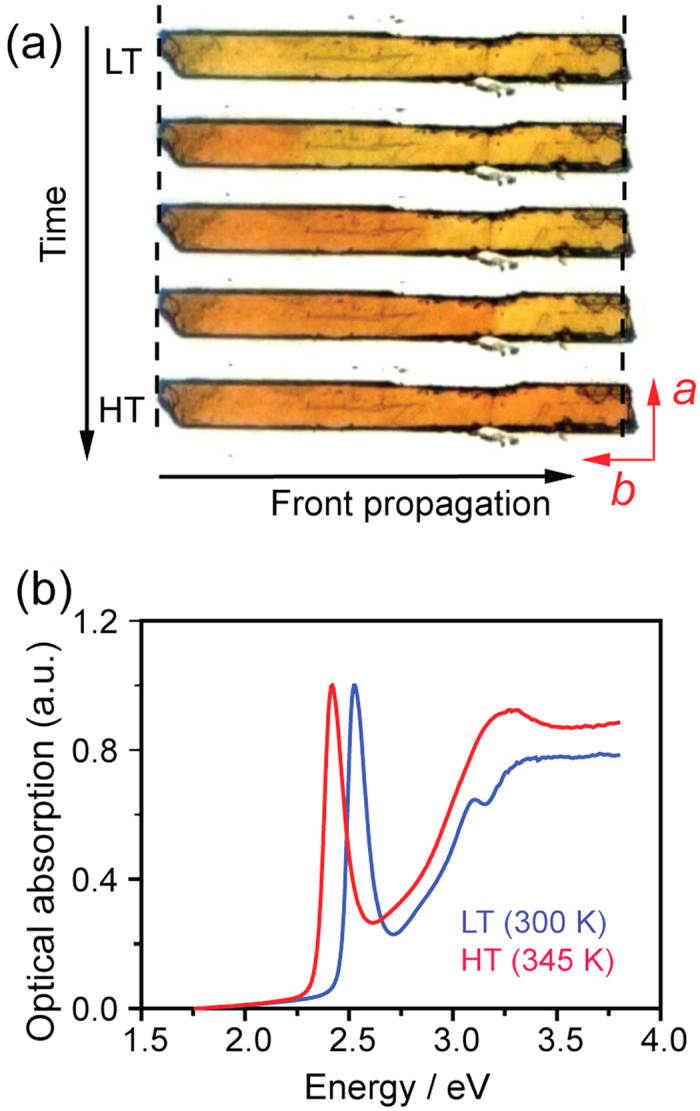
Crystal snapshots upon transition. (**a**) Selected snapshots of a C_12_PbI_4_ single crystal during the phase transition on heating. The front of the transformation appears around 317.5 K and propagates across the whole crystal within hundred milliseconds. (**b**) Optical absorption spectra of the LT and HT phases recorded on heating.

**Figure 2:**
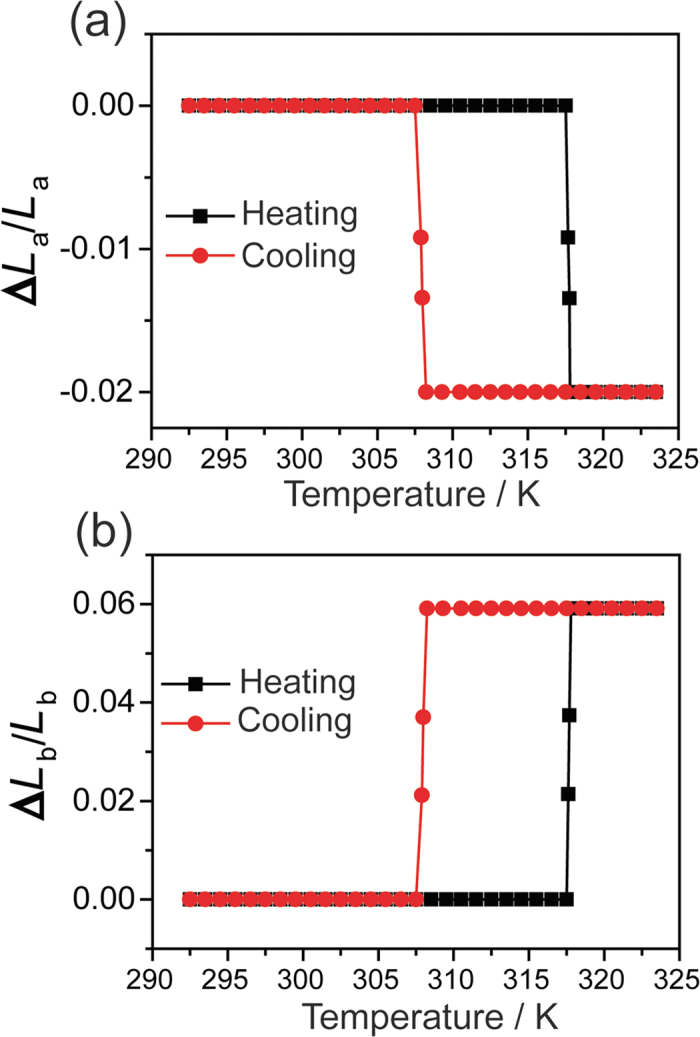
Thermal variation of the crystal size. Thermal dependence of the relative variation of the length (*L*_a_) and width (*L*_b_) of the crystal along the crystallographic axes *a* (**a**) and *b* (**b**), respectively. The crystal size was recorded during heating and cooling at temperature sweep rate 0.2 K min^−1^.

**Figure 3:**
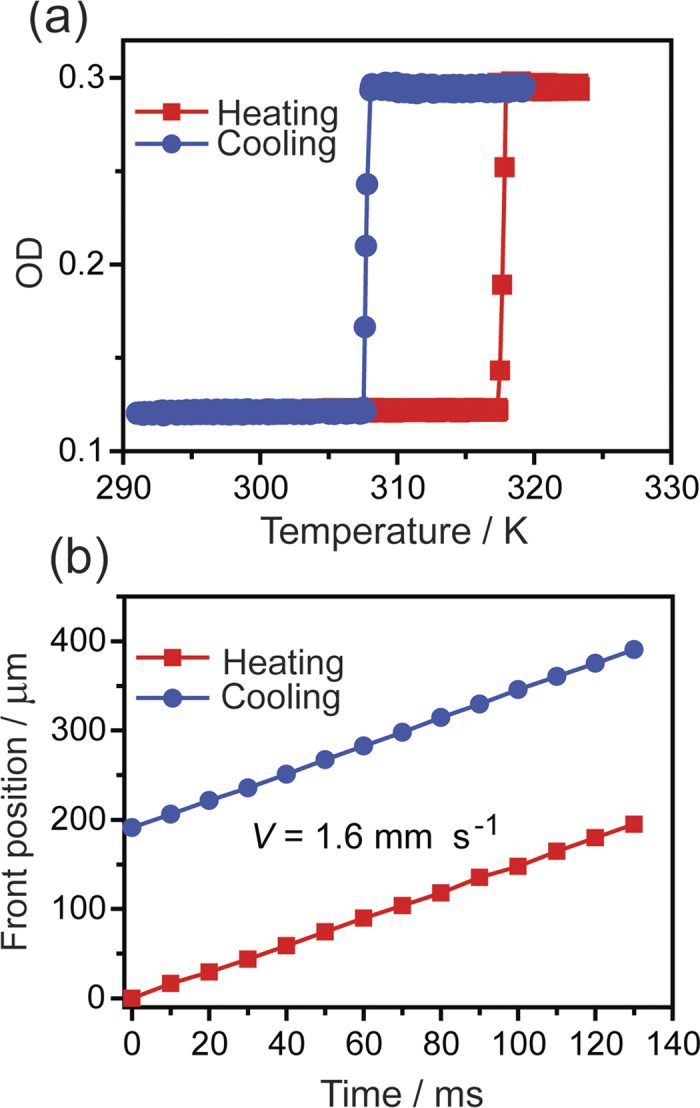
Thermal hysteresis and interface position upon phase transition. (**a**) Temperature dependence of the green optical density (OD) recorded on heating (red) and cooling (blue). The presence of a hysteresis loop with a width of 10 K confirms the first-order character of the phase transition. (**b**) Plot of the time-dependence of the front position during heating and cooling, showing a linear trend. Note that the velocity of both processes is nearly identical, *V* ≈ 1.6 mm s^–1^.

**Figure 4:**
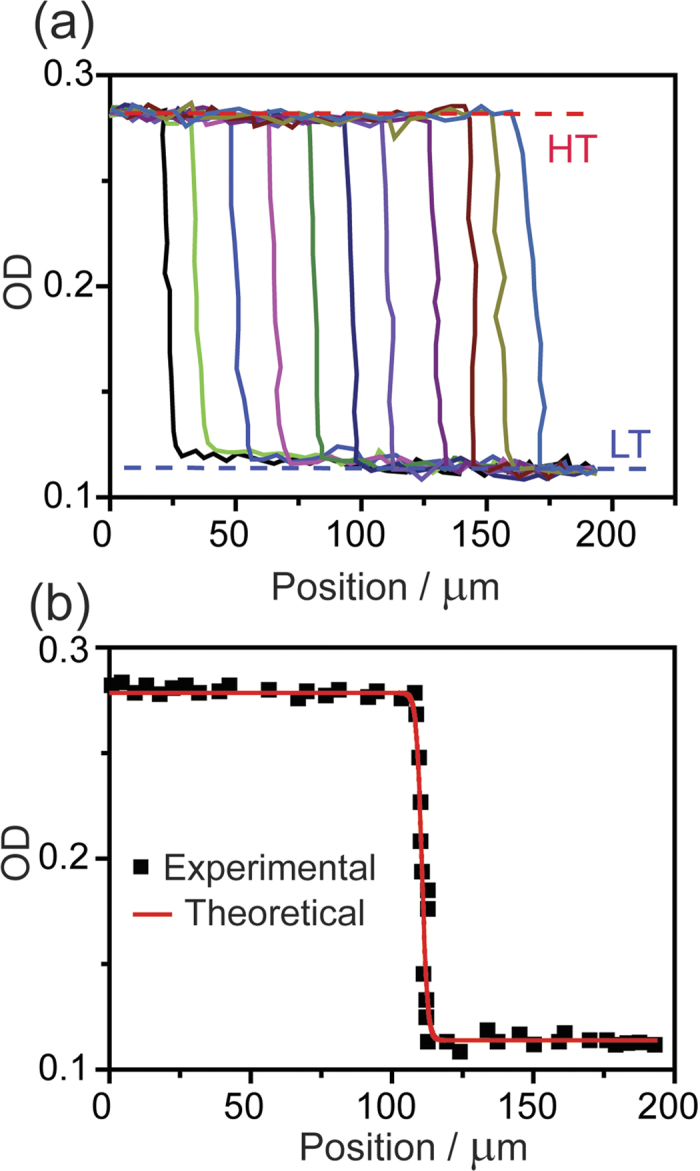
Spatial profile of the habit plane. (**a**) Profiles of the optical density (OD) recorded along the *b* axis (the propagation direction) starting from *t* = 1 s with 0.01 s time-steps between the consecutive curves, showing the uniform character of the front propagation. (**b**) Average front profile obtained by summing all data in panel a, after translation of the interface center. The red curve is the best fit obtained using equation (1).

**Figure 5:**
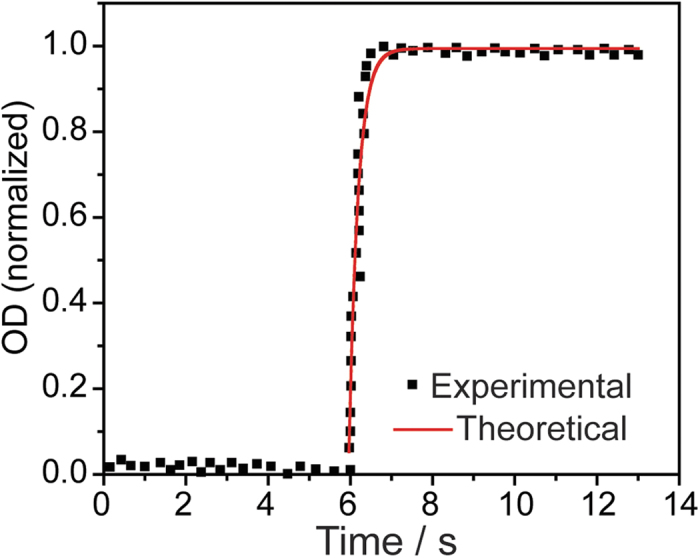
Local kinetics of the nucleation regime ahead the interface. Time-dependence of the normalized local optical density (OD) upon heating along a line perpendicular to the front propagation direction. The red curve is the best fit obtained using equation 2.

**Table 1:** Crystallographic data associated with the structural phase transition of C_12_PbI_4_^7^.

Temperature	293 K	319 K
Crystal system	Orthorhombic	Monoclinic
Space group	*Pbca*	*P*2_1_/*a*
*a*/Å	8.8645(1)	8.6882(6)
*b*/Å	8.5149(6)	9.0031(2)
*c*/Å	49.0253(9)	23.8647(8)
*V*/Å^3^	3700.45(2)	1866.71(7)
*β*/°	90	92.487(2)
*Z*	4	2
Color	Yellow	Orange

**Table 2:** Transition temperatures (*T*), mechanical resilience to the phase transition, and average interface velocities (*V*) in spin-crossover single crystals and in C_12_PbI_4_.

Crystal	*T*/K^a^	Resilience^b^	*V*/(mm s^–1^)	References
[Fe(bbtr)_3_](ClO_4_)_2_^c^	82/98	no	0.009	21
[Fe(btr)_2_(NCS)_2_] H_2_O^d^	120/145	no	0.002	22,30
[{Fe(NCSe)(py)2}2(μ−bpypz)]^e^	108/116	yes	0.010	23
Fe(bapbpy)(NCS)_2_^f^	231/237	yes	0.007	29
[Fe(ptz)_6_](BF_4_)_2_^g^	120/134	no	0.010	31
C_12_PbI_4_	310/320	yes	1.6	This work

^a^The two values correspond to cooling and heating, respectively.

^b^Preservation of the macroscopic integrity of the crystal during the phase transition.

^c^bbtr = 1,4-di(1,2,3-triazol-1-yl)butane.

^d^btr = 4,4′-bis-1,2,4-triazole.

^e^py = pyridine, μ−bpypz = 3,5-bis(2-pyridyl)pyrazolate.

^f^bapbpy = *N*,*N*′-di(pyrid-2-yl)-2,2′-bipyridine-6,6′-diamine.

^g^ptz = 1-propyltetrazole.
